# The Baltimore Academy of Medicine

**Published:** 1886-03

**Authors:** 


### THE BALTIMORE ACADEMY OF MEDICINE.

---

Officially Reported for THE ATLANTA MEDICAL AND SURGICAL JOURNAL.

STATED MEETING, January 6, 1886.

Dr. J. J. Chisolm presented the following notes on

### A PIECE OF IRIS LIVING IN THE VITREOUS CHAMBER.

G. T. S., æt. 59, six months ago, falling down a railroad embankment, received an injury to the right eye. When seen by his family physician six hours after the accident, he was suffering intensely. The lids had already become very much swollen. Upon carefully separating them a wound was discovered at the upper edge of the cornea from which a thick splinter of wood was protruding. The removal of this bit of wood was accompanied by a bloody aqueous discharge, leaving, however, so much blood in the anterior chamber as to conceal the amount of injury done to the eye contents.

The treatment pursued was rest, cold applications, and the internal administration of anodynes. As the swelling subsided and the blood was absorbed, it was found that sight had been so materially impaired as to leave only perception of light. In time, however, the vision slowly improved till after three months large objects could be again recognized. In the meantime, the left good eye indicated some growing impairment of vision for distant objects and the spectacles heretofore worn with comfort no longer permitted easy reading.

This growing defect in vision in the good eye was supposed to be a sign of sympathetic complications, and for this reason the patient was sent to me from his distant home for surgical treatment.

He complained of something moving about in his eye which seemed to wave before his sight. By oblique illumination I could see a whitish body in the vitreous. With ophthalmoscopic illumination the examination showed a healthy fundus and clear vitreous, so as to give a perfect retinal picture.

Hanging from the roof of the vitreous chamber at some little distance behind the thin, partial capsular film was a flap of membrane apparently one line wide and two lines in length. It was rectangular in shape and of a yellowish white color. Upon its anterior surface could be clearly traced a vessel of same size, which starting at the base ran down through the whole length of the membrane to the free extremity of the flap.

This piece of living tissue moved to and fro with the movements of the eye-ball. This floating membrane in the vitreous, adhering to the upper anterior edge of the choroid, could be nothing else than the missing piece of iris.

The splinter of wood, in entering the eye through the upper scleral border of the cornea, had torn the iris in two places from the pupiliary border to the ciliary region, in this way punching a piece out of it. This detached bit of iris had been pushed back into the vitreous chamber, carrying with it a portion of its ciliary body. It remained adherent to the ciliary body at one point by a broad base.

The lens must have been injured also at the time of the accident, because through its torn capsule the aqueous humor had been brought in contact with it to its complete disappearance by absorption.

As there was a broad base to the flap with ample nourishing blood-vessels, the piece of displaced iris had continued to live. Although it had been bleached and all trace of pigment had disappeared from it, it was yet thick enough to show us translucency. Under ophthalmoscopic examination it showed boldly as a whitish yellow membrane against the healthy red reflex of the fundus. Curiously the passage made by the splinter into the vitreous chamber had not been invaded by inflammatory deposits. The hyaline structures had taken on no pathological changes, and therefore excellent vision had been retained to this curiously injured eye.

Dr. J. Edwin Michael then related

### AN UNUSUAL CASE OF GUNSHOT WOUND.

On Christmas night last, about 9 o'clock, he was called to see a large, stout, well-built German man, a saloon-keeper by occupation, who had received a pistol wound while engaged in quelling a disturbence in his establishment.

The ball came from a pistol of the "bull dog" pattern, 38 calibre, and was shot from a distance of 12 to 14 feet in front of and a little to the patient's right.

Upon examining the patient it was seen that the point of entrance of the ball was at a spot almost if not directly over the apex beat of the heart.

Later measurements showed the exact location of the perforation to be three inches to the left of medium line, one and one-half inches to right of left nipple line and three-fourths of an inch below a line drawn transversely through the nipple. There was of course no probing. The patient was in a condition of extreme shock; stimulants were given and after a short while consciousness returned. Upon examination by physical signs, wound of the lower lobe of the left lung and hemorrhage into the pleural cavity of that side was diagnosed. It was also probable that there was a co-existing pneumo-thorax of small degree. About eleven o'clock, two hours after the accident, the patient began to complain of much general pain. He became quiet after a hypodermic of morphia and atropia, and continued comparatively easy for a time, when pain again began to be felt, which was again arrested by a repetition of the hypodermic injection.

At the end of 20 hours from the time of the accident the patient died.

The autopsy showed that the bullet had passed through the fifth rib, near its junction with the cartilage, through the pericardium, through the left ventricle, making in its passage an opening at its point of entrance and another at its point of exit, which two openings were connected by a furrow in the ventricular wall, ploughed up by the ball in its passage through the heart. After leaving the heart it had penetrated and passed through the lower lobe of the left lung, fractured the eleventh rib and imbedded itself in the soft tissues of the back about two inches to the left of the spinal column.

Dr. J. J. Chisolm said of course we had all heard of the rare case of perforation of the heart and retention of the bullet within its cavity until found there after death. He had seen cases of injury to the heart by bullets, but none of them had lived so long as 20 hours.

Dr. A. B. Arnold next read a most exhaustive paper on

CIRCUMCISION, in which he treated the subject from a moral as well as from a historical and surgical standpoint.

He protests against the practice, not only because he considers it detrimental in robbing the glans of its natural protective covering and thus exposing to irritation those very sensitive pacinian bodies about the corona. The result of this exposure and consequent irritation he thought led to habits of masturbation. From the religious point of view he considered it an entirely erroneous idea that a child born of a Hebrew mother was not a Hebrew until circumcision had been performed. It has recently been decided, he said, that a Jew cannot be excluded from fellowship in a Jewish congregation because of the presence of a foreskin.

Dr. Stanley Hall, who was present, was invited to make some remarks upon the subject. He said, after Dr. Arnold's paper, there was left but little for him to say—he had always been more or less in the dark on the subject, but had never had such a flood of light given him upon the topic as had been afforded by Dr. Arnold's treatise. Some few years since he was in Vienna during a discussion on the subject, and it was there decided that circumcision was beneficial in lessening the erethic habit, and that it was anticipating what nature would herself do if left to her own course.

Dr. John R. Uhler said that possibly circumcision had its effect upon procreation by lessening the amount of surface tissue to be filled with blood and thus allowing a greater amount of blood to be supplied to the center of the organ. This would, he thought, have the effect of increasing the temperature of the body of the organ. Again he thought that possibly the reason for its having been first practiced in Eastern countries, and especially in the tropics, was that the dry sandy atmosphere so potent in causing ophthalmia might have such an irritating effect by causing a gritty deposit beneath the foreskin.

Dr. J. Edwin Michael thanked Dr. Arnold for his paper. He had sometime since read an article on the subject from a surgical standpoint. In his experience he had been frequently called upon to remove the prepuce because of its inconvenience. As to affecting the sensitiveness and irritability of the glans, he did not see that the operation made any difference whatever, for how very often do we see men with naturally exposed glans from an abnormally short prepuce. They present no greater degree of sensitiveness. He thought with some eminent authorities that the sensitiveness of the glans is increased by circumcision of long prepuce.

---

CALOMEL AS A DIURETIC.—The action of calomel in causing diuresis in morbid conditions with dropsy is not generally recognized. In health, indeed, it may be said that the drug has no such action. Dr. Jendrassic has found in cases of cardiac dropsy that calomel in appropriate doses causes well-marked diuresis, a "sort of diabetes insipidus," by which the results of want of cardiac compensation, dropsy and œdema, are dissipated. The effect comes on within twenty-four hours, one and a half grain of the drug being given three to five times a day. No diarrhœa is usually produced; but in some cases it had to be prevented by the administration of laudanum. Salivation and stomatitis were obviated by the prescription of a chlorate of potash gargle from the first. The result in all cases in which the treatment was adopted was beneficial, no unfavorable depressing symptoms being noticed.—*British Medical Journal, Feb. 13.*

